# Regulatory T cells in spondyloarthropathies: genetic evidence, functional role, and therapeutic possibilities

**DOI:** 10.3389/fimmu.2023.1303640

**Published:** 2024-01-15

**Authors:** Stefano Rodolfi, Connor Davidson, Matteo Vecellio

**Affiliations:** ^1^ Department of Rheumatology and Clinical Immunology, IRCCS Humanitas Research Hospital, Milan, Italy; ^2^ Department of Biomedical Sciences, Humanitas University, Milan, Italy; ^3^ Wellcome Centre for Human Genetics, University of Oxford, Oxford, United Kingdom; ^4^ Centro Ricerche Fondazione Italiana Ricerca Sull’Artrite (FIRA), Fondazione Pisana per la Scienza ONLUS, San Giuliano Terme, Italy

**Keywords:** CD4^+^ regulatory T cells (Tregs), ankylosing spondylitis (AS), immune-mediated rheumatic disease, genetics, genomics, spondyloarthritis, transcription factors, single-cell genomics

## Abstract

Regulatory T cells (Tregs) are a very specialized subset of T lymphocytes: their main function is controlling immune responses during inflammation. T-regs involvement in autoimmune and immune-mediated rheumatic diseases is well-described. Here, we critically review the up-to-date literature findings on the role of Tregs in spondyloarthropathies, particularly in ankylosing spondylitis (AS), a polygenic inflammatory rheumatic disease that preferentially affects the spine and the sacroiliac joints. Genetics discoveries helped in elucidating pathogenic T-regs gene modules and functional involvement. We highlight T-regs tissue specificity as crucial point, as T-regs might have a distinct epigenomic and molecular profiling depending on the different site of tissue inflammation. Furthermore, we speculate about possible therapeutic interventions targeting, or enhancing, Treg cells in spondyloarthropathies.

## 1 Introduction

Ankylosing spondylitis (AS) is the prototype of a group of arthritides known as spondyloarthropathies (SpA) because of their predilection for the spine and other parts of the axial skeleton, particularly the sacroiliac joints. (SpA) (1, 2). Other than spondylitis, AS clinical spectrum encompasses the presence of enthesitis, which is the inflammation of the specialized stress-bearing interfaces between tendons/ligaments and bone known as entheses, dactylitis (the inflammation of the entire digit), and peripheral synovitis, which are variably associated with the classical clinical phenotype of AS (3). Furthermore, extra-articular manifestations of AS include anterior uveitis, inflammatory bowel disease, and skin psoriasis (2). Considering the radiographic sacroiliitis as a dominant characteristic in AS, the Assessment of SpondyloArthritis international Society (ASAS), agreed in changing the nomenclature, referring to axial spondyloarthritis (axSpA) as the overall term for the disease (both radiographic and non-radiographic axSpA) (4). For the purposes of this work, these terms will be used interchangeably.

AxSpA is a complex disorder involving, together with environmental factors, a strong genetic component. The association with HLA-B27 is the strongest ever documented in AS, but recently, >100 loci have been significantly incriminated by genome-wide association studies (GWAS) (5).

Back in 2013, the Immunochip study by the International Genetics of Ankylosing Spondylitis (IGAS) consortium has been crucial in highlighting the role of the immune system in AS, discovering association for several immune-specific genomic loci with increased risk in developing the disease (6). Many of these genes were involved in influencing T-lymphocyte activation and differentiation. The best example is the interleukin-23 receptor (*IL23R*), crucially involved in Th17 immune responses, which, in physiological conditions, contributes in maintaining the integrity and pathogen clearance at mucosal surfaces but becomes overactivated in AS (6) (7). The importance of the IL23R pathway in the disease is also strengthened by the association with AS of *TYK2* (tyrosine kinase 2), *IL27*, and *IL6R*, genes with known effects on the IL-23 pathway (6). In particular, IL-6 signaling, through IL-6R, together with transforming growth factor (TGF)-β, influences the ratio of specific subsets of specialized T lymphocytes, in particular Th17 cells to regulatory T-cells (Tregs), promoting the differentiation of Th17 from naive T cells and inhibiting TGF-β–induced differentiation into Treg cells (8). Tregs are a specialized subset of T lymphocytes, and they are crucial in the maintenance of immunological self-tolerance and homeostasis (9, 10).

The conversion of Tregs to a dysfunctional, pathogenic, and pro-inflammatory phenotype is observed in a plethora of immune-mediated diseases, including axSpA (11). The role of Tregs in axSpA, including molecular and genetics mechanisms acting on their stability and suppressive capacity, specialized features, and functional pathways, will be discussed in this review, with a view on the possibility of developing effective therapeutics.

## 2 The genetic bases of Treg dysfunction in ankylosing spondylitis

In 2015, in their seminal work, Ellinghaus and colleagues investigated the genetic landscape of five chronic inflammatory diseases, including AS (5). The authors showed the complex pleiotropy and the relationship between these clinically related disorders (AS, Crohn’s disease, psoriasis, primary sclerosing cholangitis, and ulcerative colitis) revealed new associations and pathogenic genes modules and highlighted the crucial contribution of immune cells subsets in these disorders.

In axSpA, the role of effector/type 17 immunity is widely recognized, although the impact and phenotype of Tregs are largely unknown (12).

Very interestingly, in Ellinghaus et al., the authors identified a missense polymorphism (*rs2236379*) for Crohn’s disease, located at exon 9 of *PRKCQ* gene encoding for the protein kinase C-theta (PKC-θ). This protein kinase is an essential component of the intracellular signaling cascades leading to the activation of proinflammatory and pro-apoptotic molecules such as activator protein 1 (AP-1), nuclear factor kappa-light-chain-enhancer of activated B cells (NF-κB), and nuclear factor of activated T cells (NFAT) (13). The activation of conventional T cells upon T-cell receptor stimulation critically depends on PKC-θ, and it is dispensable for Treg-mediated suppression (14).

It has been further demonstrated that PKC-θ inhibition enhances Tregs function and protects Tregs from inactivation by TNF-α. In addition, this mechanism restores the activity of defective Tregs from rheumatoid arthritis patients and enhances protection in inflammatory colitis mice (14).

Recent works have shown the possibility to consider AS as a T-lymphocyte-associated disease: different CD4+ T cells subsets may participate in the development of AS (15, 16) (17). Many studies suggested that forkhead/winged helix transcription factor P3 (FoxP3)-positive regulatory T cells (Tregs) might play a role in the etiology of the disease.

The expression of the master transcription factor FoxP3 and the interleukin (IL)-2 receptor α-chain CD25 are the hallmarks of Tregs, as demonstrated in 2003 in a seminal paper by Sakaguchi and colleagues (18). Traditionally, based on the differential expression of three genes, FOXP3, CD25, and CD45RA, three different Tregs fractions are generally described. The first fraction is associated with CD45RA^+^FOXP3^low^/CD25^low^ and represents the resting or naive Tregs. The second fraction is associated with CD45RA^−^FOXP3^high^/CD25^high^ expression, and it defines the effector Tregs, with immunosuppressive functions. The third and last fraction is defined by CD45RA^−^FOXP3^low^/CD25^low^ cells and represents a population of cytokine-secreting Tregs, with low to none immunosuppressive capacity (19).

The complex process of cell differentiation is characterized by the formation of the so-called “epigenetic landscapes”, together with specific transcriptional programs that control the fate of definite cell types (20). Establishing the importance of FOXP3, a Treg cell-specific DNA hypomethylation pattern is also fundamentally required to establish a proper development of Treg cells that can control excessive immune cell reactivity (21). Appel and colleagues investigated the DNA methylation pattern within the Treg-specific demethylated region (TSDR) within the Foxp3 locus and demonstrated that most of the CD4+CD25+CD127− T cells exhibited demethylated TSDR, and this correlated with stable Foxp3 expression and Treg function (22). In addition, it is essential that a set of super-enhancers (multiple enhancers in close genomic proximity) (23) is established during early thymic development of Treg cells to activate a proper Tregs gene signature. Failure to activate these super-enhancers leads to impairment of the formation of Treg cells and contributes to autoimmune diseases in mice (24).

**Figure 1:**
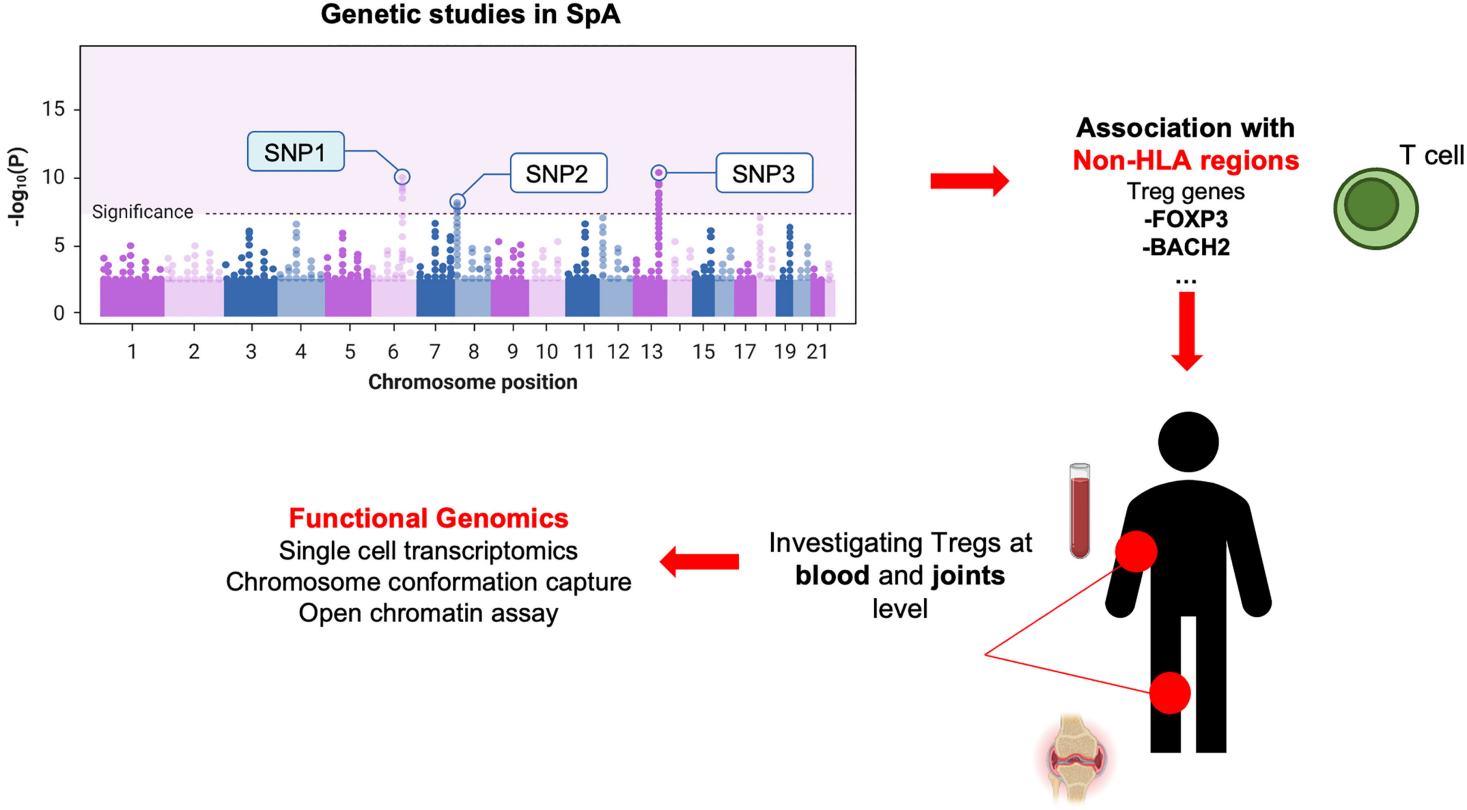
Genetics-driven approach to study Tregs in SpA. This figure illustrates the connection between GWAS findings of causal SNPs in non-coding regions of immune cell (i.e., enhancers) and the expression of Tregs genes. This approach highlights Tregs’ potential contribution as causal mediators or ineffective regulators in axSpA (or other immune-mediated/autoimmune disorders), emphasizing the genetic underpinnings of their involvement.

Overall, the evidence stemming from GWAS findings with the identification of causal SNPs for common autoimmune diseases located at non-coding regions of immune cell enhancers, associated with the expression of Tregs (25), suggests a substantial contribution of Tregs to autoimmune disorders and immune-mediated rheumatic diseases (**Figure 1**), either as potentially causal mediators or as ineffective regulators.

## 3 Tregs gene signature and modules

The impact of Tregs as mediators of inflammatory response is crucial in autoimmune diseases (26). A decade ago, a seminal study by Komatsu and colleagues evaluated the *in vivo* stability of *Foxp3* gene in Foxp3^+^CD4^+^ T cells and its impact on a mouse model of collagen-induced arthritis (CIA). The authors elegantly demonstrated that Th17 cells originating from Foxp3^+^ T cells have a key role in the pathogenesis of autoimmune arthritis (27). In specific, CD25^low^Foxp3^+^CD4^+^ T cells lose Foxp3 expression under arthritic conditions and trans-differentiated into Th17 cells, accumulating in the inflamed joints. The authors demonstrated a high level of CpG methylation for *Foxp3*, while *IL2ra* and *CTLA4* (cytotoxic T-lymphocyte-associated antigen 4) were partially methylated (27). This work confirmed the instability of Foxp3 as crucial factor in the generation of pathogenic Th17 cells in a mouse model recapitulating inflammatory arthritis.

Nevertheless, despite the importance of FOXP3, this factor alone is not sufficient to specify the complete Treg cell transcriptome (28), as what occurs in human conventional T cells expressing FOXP3, which lack suppressive capability. FOXP3 chromatin immunoprecipitation experiments followed by sequencing (ChIP–seq) in Tregs showed that only a small pool of genes that are dependent on intact FOXP3 expression are bound by FOXP3 (29, 30) either in humans or in mice, suggesting that only a small fraction of the Treg transcriptional program is directly regulated by FOXP3 (31).

Arvey and colleagues characterized the gene regulatory elements of Tregs with conserved lineage-specific activity in mice and humans, defined as increased or decreased levels of acetylation of the lysine K27 of the histone 3 (H3K27ac). The authors identified conserved epigenetic modifications in a small set of regulatory gene elements, including *Foxp3*, *Il2ra*, *Ctla4*, *Ikzf2* (Helios), *Blimp1* (B-lymphocyte-induced maturation protein 1), and *Lrrc32* (leucine rich repeat containing 32), which act as a functionally important marker of activated Tregs in human (32).

Among these, T-cell activation-associated genes *IL2RA* and *CTLA4* are of importance as several autoimmune disease-associated genetic variants have been found to localize in non-coding enhancer regions of these genes (21).

The core of a network of accessory and lineage-specifying transcription factors that is necessary to imprint Treg cell specification, maturation, and function is constituted by different members of the basic helix–loop–helix family (AHR); the basic leucine zipper (bZIP) family (in particular BACH2, NFIL3, and BATF); the zinc-finger domain family, such as BLIMP1, Ikaros and BCL-11B; the CUT homeobox family (SATB1); and the interferon regulatory factor family (IRF4).

The Ikaros zinc-finger transcription factor family has been extensively associated with Treg cell biology (33, 34). For example, IKZF2 (35) and IKZF4 (34) (*Helios* and *Eos*) exhibit their expression peaks at the Treg precursor stage, prior to FOXP3 induction. Furthermore, it has been shown that the conditional deletion of IKZF1 (*Ikaros)*, a repressor of pro-inflammatory genes (36), in CD4^+^ T cells, is responsible in the impairment of Tregs differentiation and promotes Th17 cell-mediated autoimmunity.

Of note, the impact of Ikaros zinc finger transcription factor family has been linked with different autoimmune disorders such as systemic lupus erythematosus and Sjogren syndrome (37).

In addition, the transcriptional activator and repressor BACH2 (BTB domain and CNC homology 2 factor) plays a critical role in the differentiation of Tregs. It has been demonstrated that *Bach2*-deficient mouse is able to develop spontaneous lymphocytic and macrophagic inflammation specifically at the gut and the lung sites, leading to death. *Bach2* ^−/−^ mice are deficient in Tregs, and their residual Treg cell niche is low in FOXP3 and does not prevent transfer colitis. Furthermore, these cells show spontaneous activation and produce an elevated range of Th1 cytokines and Th2 cytokines. The increased positivity for antinuclear and anti-double-stranded DNA autoantibodies in *Bach2* ^−/−^ mice confirmed its functional involvement in autoimmune disorders (38).

Another example is NFIL3 (nuclear factor, interleukin 3 regulated), which has a multifaceted role in regulating numerous biological processes, and it is a negative regulator of FOXP3 expression. In the immune system, NFIL3 regulates plasticity and cytokine production of Th1, Th2, and Treg cells. Tregs express the lowest NFIL3 level: it has been demonstrated that NIFL3 overexpression attenuates the suppressive ability and stability of Tregs (39). It is remarkable that NFIL3 binds directly the promoter of *Foxp3*, downregulating its expression and also the expression of other Tregs master genes such as *ICOS* (inducible T-cell costimulator), *CTLA4*, and *IL12RA*, all fundamental members of the Tregs gene regulatory network.

**Figure 2:**
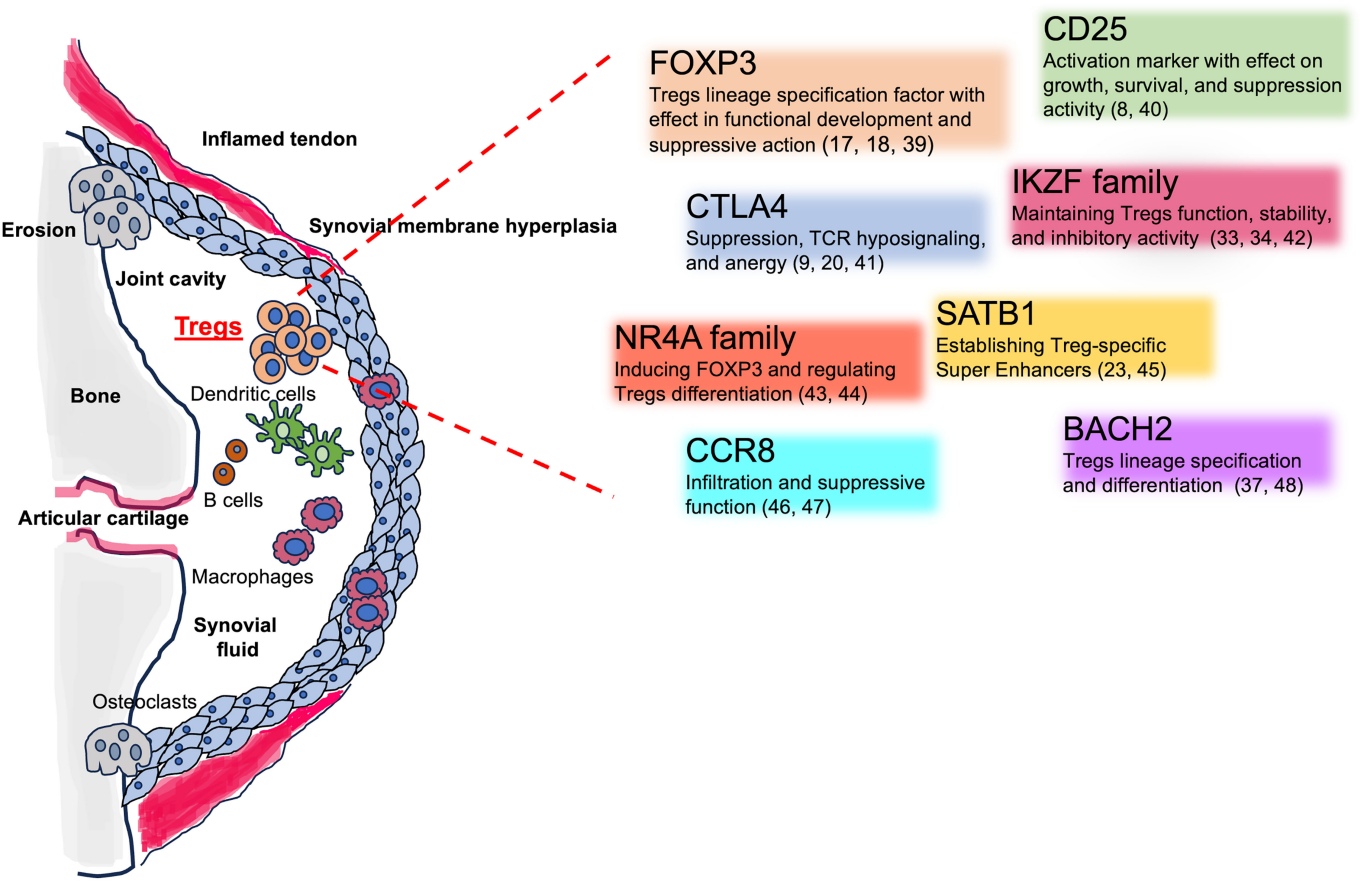
Tregs involvement in the SpA inflamed joints and Tregs key associated genes. The figure shows the different cellular players in in the inflamed joints, including Tregs. The cartoon encloses Tregs-associated genes, their mechanisms of action, and key references.

The heterogeneity of Tregs manifested by differential gene expression (see **Figure 2**), trafficking, and functional properties makes these cells a complicated matter to study: niche specificity is crucial when approaching this topic.

## 4 The importance of studying Tregs at the inflamed site: the synovia and the concept of niche specificity

It is essential to investigate the phenotypic and transcriptional profile of human Tregs in the different sites of inflammation or in autoimmune-injured tissues (8). Understanding Tregs features in different tissues is crucial for determining the regulatory mechanism of maintaining homeostasis (40). Unfortunately, studying Tregs at the sites of inflammation during AS is difficult, considering the limited accessibility to the organs affected for clinical and investigational sampling (41). For this reason, the only relevant inflammatory site sampled with the aim of characterizing Tregs has been the synovial fluid during a peripheral arthritis flare. Although peripheral arthritis is questionably not an AS-specific manifestation, the investigation of Tregs in the synovia represents a good opportunity to study the local regulatory mechanisms of immune-mediated arthritis.

During inflammation, T-cell trafficking towards peripheral organs relies on a complex network of cytokines, integrins, chemokines, and their corresponding receptors. Specific trafficking pathways can cause imbalanced ratios of cell subpopulations. In the case of Tregs, selective recruitment, changes in frequency, or an increased influx of conventional effectors able to outnumber Tregs are all scenarios that could explain the disruption in homeostasis. Nevertheless, *in vivo* and *ex vivo* data indicate that Tregs are actively recruited to the sites of inflammation and participate in the inflammatory process of inflammatory arthritis (42). A recent work examining joint changes in an RA mouse model confirmed the importance of niche-specificity investigation, as the authors demonstrated that the activation of macrophages in the synovial lining niche starts the recruitment of neutrophils and initiates articular inflammation, shifting the paradigm considering the lining macrophages as immunosuppressive cells into possible troublemakers (43).

Synovial fluid and peripheral blood are the most common and accessible samples to study Tregs phenotype and function in axSpA. Cao and colleagues showed an enrichment in the number of Tregs in the synovial fluid of AS patients (44). Furthermore, a higher frequency of Foxp3^+^ cells/CD4^+^ T cells within synovial fluid compared to peripheral blood was observed, compatible with a Tregs-suppressive phenotype (22). Unfortunately, there are a lot of controversies throughout the years in evaluating the changes in the frequency of Tregs in synovial fluid and peripheral blood. Increase, reduction, or no changes have been reported in the number of circulating Tregs, and this is mainly due to differences in choosing the right surface-specific Tregs markers among the authors (45).

Recently, thanks to single-cell transcriptomic analysis, it has been possible to uncover population heterogeneity and to identify smaller sub-populations of interest.

In 2022, Simone and colleagues investigated the single-cell transcriptome of >16,000 Tregs obtained from peripheral blood and synovial fluid in two HLA-B27^+^ AS patients and three patients with an associated spondyloarthritic condition and psoriatic arthritis.

The authors have identified multiple Tregs clusters: among these, one regulatory CD8^+^ subset expressed cytotoxic markers/genes, while a Th17-like *RORC* ^+^ Treg subset was characterized by the expression of IL-10 and lymphocyte activating 3 (LAG-3, which plays an important immunoregulatory role) like a subset described in other inflammatory conditions (46).

Single-cell RNA sequencing technology was also used in another recent work showing that a subset of Tregs significantly increased after anti-TNF treatment and negatively correlated with BASDAI (Bath Ankylosing Spondylitis Disease Activity Index). This cluster was characterized by the strong expression of *CTLA4, TGFB1*, *CD25*, and *CD279*, suggesting a highly suppressive phenotype (47).

The investigation of Tregs on a specific site of inflammation, although at early stages, holds promise, as it can reveal the high level of heterogeneity and describe the adoption of specific transcriptional programs and the use of preferred effector pathways that could be used therapeutically.

There is much evidence that indicates functional defects in Tregs in axSpA. For example, it has been demonstrated that Foxp3 Treg cells isolated from axSpA patients showed a downregulation in T-cell immunoglobulin and mucin-domain containing molecule 3 (Tim-3) expression. Tim-3 is a transmembrane protein expressed on Tregs surface, and it is associated with stronger suppressive capacity. This Tim-3^+^ Tregs subset exhibited a higher expression of IL-10, granzymes, and perforin, and a higher Foxp3 expression than Tim-3^−^ Tregs. A lower percentage of Tim-3^+^ Tregs in axSpA patients than in healthy controls indicates the role of dysfunctional Tregs in the disease’s pathophysiology (48).

As described, a deeper investigation of Tregs in axSpA tissues is crucial to move the field forward, as Tregs gene expression, metabolic pathways, and functional variation are strictly related to tissue specificity (see **Table 1**).

**Table 1:** Different features of synovial fluid Tregs comparing axSpA, OA, and RA.

Tregs	axSpA	OA	RA
Number	Synovial fluid >peripheral blood	Synovial fluid >peripheral blood	Synovial fluid >peripheral blood
Phenotype	CD45RA−CD25+CD127^low^	CD4^+^CD25^+/high^CD127^−/low^	CD4^+^CD25^+/high^CD127^−/low^
Features	FOXP3+TIGIT+CD27+ most abundant cluster	Characterized by IL-10 production	Expression of higher levels of CTLA-4, GITR, and OX40
	Upregulation of interferon signature and TNF receptor superfamily genes	Presence of CD45RO^+^RA^−^ memory phenotype	Presence of CD45RO^+^RA^−^ memory phenotype
	Clonal expansion in synovial fluid with distinct transcriptional features (upregulation of CD177, SIRPG, S100A6, and S100A4) (46)	Specifically synovial Tregs are activated effector memory cells (CD62L-CD69+), with increased CD152 expression (49)	Increase in Tregs expressing a high level of RANKL in RA synovial tissue samples (50)

*axSpA, axial spondyloarthritis; OA, osteoarthritis; RA, rheumatoid arthritis.

## 5 Tregs and treatment for AS

Evidence on the important role of Tregs in the pathophysiology of axSpA is added by the changes observed after pharmacological treatment. Yang et al. reported a baseline imbalance of peripheral Th17 cells over Tregs in favor of the former in AS patients compared to healthy controls, which was then restored after TNF-alpha inhibition, which represents a cornerstone treatment for AS. Moreover, this was accompanied by an increase in B10 cell count (B cells secreting IL-10, a subclass of B regulatory cells). The increase in Tregs frequency inversely correlated with the levels of C-reactive protein (51). Furthermore, a large study on 222 patients with active AS confirmed Tregs increase after anti-TNF therapy, with a negative correlation with BASDAI score. Increase in Tregs levels was accompanied by a decrease in proinflammatory cytokines such as TNF-alpha, IL-6, IL-17, and IL-23, while concentrations of TGF β increased. Interestingly, all these changes were detected only in patients who showed a clinical response to TNF blockade, while non-responders did not show an increase in peripheral Tregs concentrations and had instead a significant increase in IL-17 and IL-23 (52). Altogether, this gives indirect evidence that when inflammation is driven by TNF-α, direct cytokine inhibition relies, with other mechanisms, on the restoration of the Tregs compartment to curb the inflammatory response. However, a recent study by Liao et al. documented opposite findings: the authors showed an increased levels of Tregs in patients with active AS, positively correlated with BASDAI and levels of proinflammatory cytokines, which were then lowered by TNF inhibition. The increased peripheral levels of Tregs was justified as a positive feedback in the attempt to curb the ongoing proinflammatory response (53).

Altogether, Tregs seem to actively participate in the pathophysiology of axSpA, and their suppressive role seems to be restored by anti-TNF therapy in patients that achieve disease remission. Further functional data are needed to support this hypothesis and to test changes in Tregs levels and function after blockade of other cytokines, such as IL-23 or IL-17.

In recent years, there have been attempts to exploit therapy with IL-2 to expand the Treg population to treat autoimmune diseases, with promising results (54). Interleukin 2 has pleiotropic effects on immune cells; it works by blocking the conversion of CD4-naive T cells into Th17 cells and by stimulating proliferation of Tregs (55–57). In 2019, a prospective, open-label, phase I–IIa study tested the efficacy of low-dose IL-2 in 46 patients with several autoimmune diseases, among which were 10 patients with active AS (58). Low-dose IL-2 had a good safety profile and proven biological efficacy by resulting in the expansion of the Treg population independently of the disease. Moreover, AS patients showed a decrease in BASDAI score and in clinical global impression scale (CGI), even though the study was not designed to test clinical outcomes.

## 6 Conclusions and future therapeutic perspectives

Since Tregs possess a wide spectrum of suppressive mechanisms and can induce tolerance upon transfer in numerous models, they have been considered for adoptive cell therapy and tested in early-phase clinical trials. Polyclonal Treg cell therapy has demonstrated its feasibility, safety, and potential efficacy in managing immune-driven conditions (59, 60). Moving forward, gene editing and bioengineering are currently being used to develop antigen-specific Tregs, which represent the next generation of Treg therapy. These cells express a chimeric antigen receptor or homing receptors, which could facilitate their trafficking to sites of organ autoimmunity (61).

In parallel, studying Tregs at the single-cell level and within different inflammatory sites will increase the knowledge on their biology for translational applications. The characterization of the Tregs transcriptional program in AS-specific inflammatory sites (i.e., enthesis, synovium, and gut) and their preferred effector pathways in these contexts remains largely unexplored areas. For instance, compelling evidence suggests that Tregs could adopt specific mechanisms, such as the regulatory immune receptor TIGIT (T-cell immunoreceptor with Ig And ITIM domains) (*in vitro*) (62) or IL-10 (63) during experimental colitis, to inhibit Th17 responses. Identifying differentially expressed effector pathways in the various inflammatory niches could open novel opportunities for experimentation, particularly in the context of significant therapeutic unmet needs.

TIGIT might represent an amenable therapeutic target. Kojima et al. recently described how anti-human-TIGIT agonistic antibodies in mouse suppressed the activation of CD4+ peripheral and follicular helper T cells and enhanced the suppressive action of Tregs, both *in vitro* and in murine models, a strategy that has been speculated to be proficient in the treatment of autoimmune diseases such as systemic lupus erythematosus and RA (64).

Leveraging Treg-mediated mechanisms of suppression for therapeutic benefit has already proven effective in RA and psoriatic arthritis, as demonstrated by the success of abatacept, a CTLA-Ig fusion protein that inhibits T-cell activation by preventing the delivery of costimulatory signals by antigen-presenting cells (65). Similar strategies could be applied to other immune-mediated conditions. For example, enhancing the function of LAG-3+ Tregs (a promising cancer immunotherapy strategy (66),) could find an application in AS and inflammatory bowel disease, where should the *in vitro* findings be confirmed, LAG-3 would emulate a natural inhibitory mechanism that hinders T-cell costimulatory signals, leading to the modulation of effector responses and suppression of Th17-mediated inflammation (46). However, further functional evidence needs to be provided, as the putative suppressive effect of LAG-3+ Tregs has not been demonstrated in the synovial or entheseal environment yet (67). Finally, the cytotoxic anti-LAG-3 chimeric antibody (A9H12), which depletes LAG-3-expressing cells, has produced promising results in a phase I/Ib, double-blind, placebo-controlled clinical study for psoriasis, showing improvement in psoriasis disease activity and downregulation of proinflammatory genes (68).

We have highlighted a high number of reports showing that Tregs participate in the etiology of AS or axSpA. However, as also previously stated in paragraph 4, there are a lot of controversies on Tregs in AS patients, particularly regarding the percentage of circulating cells (44, 69). Increase, reduction, or no changes in the percentage of circulating Tregs have been reported in axSpA patients, making the topic challenging (45, 70).

In conclusion, it is of utmost importance to understand the context of Treg molecular mechanisms of suppression to boost cell-based therapies and identify novel amenable targets to enhance or restore these mechanisms when deficient. In the context of AS and spondyloarthropathies, a crucial focus would be to determine which Treg mechanisms are most effective in controlling Th17 responses. Conventional *in vitro* cell-based suppression assays frequently provide only limited insights into the intricate cellular cross-talk involved, making it pivotal to uncover more robust and relevant mechanisms for effective therapeutic interventions.

## Author contributions

SR: Conceptualization, Writing – original draft, Writing – review & editing. CD: Writing – original draft, Writing – review & editing. MV: Conceptualization, Writing – original draft, Writing – review & editing, Supervision.
